# Immune Escape in Glioblastoma: Mechanisms of Action and Implications for Immune Checkpoint Inhibitors and CAR T-Cell Therapy

**DOI:** 10.3390/biology12121528

**Published:** 2023-12-15

**Authors:** Catherine Yu, Kristin Hsieh, Daniel R. Cherry, Anthony D. Nehlsen, Lucas Resende Salgado, Stanislav Lazarev, Kunal K. Sindhu

**Affiliations:** Department of Radiation Oncology, Icahn School of Medicine at Mount Sinai, New York, NY 10029, USA; catherine.yu@icahn.mssm.edu (C.Y.); daniel.cherry@mountsinai.org (D.R.C.); anthony.nehlsen@mountsinai.org (A.D.N.); lucas.resendesalgado@mountsinai.org (L.R.S.); stanislav.lazarev@mountsinai.org (S.L.)

**Keywords:** glioblastoma, tumor microenvironment, immune checkpoint inhibitors, chimeric antigen receptor (CAR) T-cell therapy, immunotherapy resistance, immunosuppression, tumor immune escape

## Abstract

**Simple Summary:**

Glioblastoma has been recognized as one of the most aggressive and fatal human tumors. Despite immunotherapy’s recent success in improving outcomes in a number of cancers, glioblastoma remains largely resistant to this treatment modality and current treatments generally fail in achieving long-term disease control. This review focuses on elucidating the unique mechanisms of immune escape that glioblastoma employs to circumvent current immunotherapy strategies. In particular, glioblastoma expertly disarms innate and adaptive aspects of host immunity, thus rendering immune checkpoint inhibitors (ICIs) and chimeric-antigen receptor (CAR) T-cell therapy largely ineffective on both local and systemic levels. These mechanisms include the secretion of immunomodulatory factors that deplete T-cell numbers and activity, an extensive tumor vasculature, and significant intratumoral antigenic heterogeneity. We also highlight promising results from preclinical studies that have directly addressed these mechanisms of immunosuppression in glioblastoma and may thus hand immunotherapy an edge in treating this devastating tumor.

**Abstract:**

Glioblastoma, the most common primary brain cancer in adults, is characterized by a poor prognosis and resistance to standard treatments. The advent of immunotherapy has revolutionized the treatment of several cancers in recent years but has failed to demonstrate benefit in patients with glioblastoma. Understanding the mechanisms by which glioblastoma exerts tumor-mediated immune suppression in both the tumor microenvironment and the systemic immune landscape is a critical step towards developing effective immunotherapeutic strategies. In this review, we discuss the current understanding of immune escape mechanisms in glioblastoma that compromise the efficacy of immunotherapies, with an emphasis on immune checkpoint inhibitors and chimeric antigen receptor T-cell therapy. In parallel, we review data from preclinical studies that have identified additional therapeutic targets that may enhance overall treatment efficacy in glioblastoma when administered alongside existing immunotherapies.

## 1. Introduction

Glioblastoma, the most aggressive type of glioma, accounts for 49% of malignant primary brain tumors [1]. Glioblastoma is highly likely to recur and carries a grim prognosis, with a median survival of less than 2 years [2,3]. 

Under the fifth edition of the World Health Organization (WHO) Classification of Tumors of the Central Nervous System, tumors are graded based on a combination of their molecular abnormalities and histological features [4]. The emerging role of molecular diagnostics has identified distinct biomarkers that accurately correlate with prognosis, with glioblastoma being associated with IDH-wildtype status, EGFR amplification, TERT promoter mutation, and concurrent gain of chromosome 7 and loss of chromosome 10 [4].

While significant progress has been made in the molecular profiling of these tumors, treatment advances for treating glioblastoma have remained sparse. The current treatment paradigm for glioblastoma, consisting of maximal safe resection followed by adjuvant chemoradiation (RT) with concurrent temozolomide (TMZ) +/− alternating electric field therapy, fails to deliver long-term disease control [3]. As a result, glioblastoma remains incurable, and thus new approaches for its management are urgently needed.

This unmet clinical need has prompted researchers to investigate the application of immune checkpoint inhibitors (ICIs) and chimeric antigen receptor (CAR) T-cell therapy to glioblastoma, as these modalities have yielded remarkable improvements in the management of several previously intractable tumors, including melanoma [5,6,7] and non-small-cell lung cancer (NSCLC) [8]. However, emerging evidence suggests that glioblastoma may be especially adept at evading the immune system, and initial clinical trials of these two immunotherapeutic approaches have been disappointing [9,10,11,12,13]. Therefore, understanding the specific mechanisms of immunosuppression in glioblastoma is critical to facilitate the effective employment of immunotherapy against this devastating tumor.

In this review, we summarize the current understanding of immune escape mechanisms in glioblastoma both in the local tumor microenvironment (TME) and in the systemic setting. We discuss how downstream effectors of tumor-driven immune suppression directly limit the utility of immunotherapy in treating glioblastoma. Additionally, we highlight emerging targets to consider alongside existing immunotherapy strategies that have shown promising pre-clinical results. 

## 2. Overview of Immunotherapy

Immunotherapy represents a new horizon in cancer treatments and a significant departure from the previous systemic treatment paradigm. Traditional chemotherapy acts directly on cancer cells through various cytotoxic effects. In contrast, immunotherapy acts indirectly on tumors by modifying key intercellular interactions: specifically, the way that the body’s immune cells interface with cancer cells [14].

Immunotherapy exploits the tendency of all cells to display surface proteins that convey important information about cells’ intracellular activity [15]. These displays, whether physiologic or pathologic, are then subject to surveillance by the body’s immune cells. Sufficiently problematic displays capture immune cells’ attention and may prompt the immune cells to earmark cancer cells for eventual elimination. However, immune cells must process these cancer signals in the context of many other intercellular signals, and cancer cells have a propensity for muddying the waters by simultaneously presenting immune-quieting signals that act on immune cell targets, called “checkpoints”. These checkpoints are inhibitory signaling pathways which, when activated, promote a pause in the immunogenicity of T cells that would otherwise move to proliferate in the presence of these danger signals. Therefore, interfering with the tumor–immune cell communications that promote checkpoints has the goal of shifting toward anti-tumor activity. 

Anti-cytotoxic T-lymphocyte-associated protein 4 (CTLA4) and anti-programmed death-1 (PD1) antibodies are two widely used, representative ICI agents [16]. In advanced melanoma [17], renal cell carcinoma [18], and NSCLC [19], seminal trials in ICI have shown overall survival benefits of 3.7 months (*p* < 0.001), 5.4 months (*p* = 0.002), and 7.1 months (*p* = 0.01), respectively. These trials were conducted in patients with advanced disease and without excellent treatment options, a situation that closely parallels the challenges experienced by patients with glioblastoma, where outcomes have been without significant improvements for decades. Unfortunately, high-profile trials investigating ICIs for glioblastoma have so far been disappointing [11,12,13].

CAR T-cell therapy represents another contemporary paradigm in immunotherapeutic approaches. Unlike ICIs, which silence inhibitory counter-messaging when immune cells have identified likely cancer cells, CAR T-cell therapy is designed to improve recognition of tumor cells [20]. Rather than relying on immune cells’ preexisting tumor recognition hardware, patient T cells are modified in the lab to carry synthetic tumor identification receptors, which in turn should respond robustly to the presence of tumor antigen [21]. This technology has demonstrated efficacy in hematologic malignancies like B-cell lymphomas and multiple myeloma, resulting in six FDA approvals since 2017 [22,23,24,25]. Research is still ongoing to elucidate the application of CAR T-cell therapy in solid tumors, where preclinical studies have shown promise, but no large, randomized studies have yet been published. The dire need for improvement in glioblastoma outcomes has prompted exploratory efforts in CAR T-cell therapy, but early pilot studies of 3–10 subjects have not provided much optimism [9,10].

Elucidating the unique immune escape mechanisms in glioblastoma may explain immunotherapy’s poor performance in these patients. With respect to ICIs, removing a brake placed on the immune system by tumors is ineffectual if the immune system itself is inherently anergic or in poor working condition. CAR T-cell therapy, which requires a key downstream player in the anti-tumor immune response, may be equally hamstrung by the dual local and systemic immunosuppression in patients with these tumors. Despite being engineered for tumor targeting, CAR T cells cannot function in a vacuum and glioblastoma employs a series of immune escape mechanisms that may blunt the CAR T cells’ engineered immunogenicity to render them ineffective [26,27]. If the full potential of immunotherapy is to be realized in glioblastoma treatment, it is necessary to apprehend and obviate the mechanisms that these tumors employ to circumvent the immune system.

## 3. Glioblastoma Achieves Resistance to Immune Checkpoint Inhibitors with Unique Immune Escape Mechanisms

### 3.1. ICI Efficacy Is Predicated on Functional Innate and Adaptive Immunity

The integrity of the host’s inherent innate and adaptive immunity—on both local and systemic levels—is critical for achieving a meaningful response to ICIs, which function by enhancing preexisting antitumor immune activity. Innate immunity initiates a non-specific, antigen-independent immune response; the cells that execute this initial defense mechanism are distinct myeloid lineage cells such as dendritic cells (DCs), macrophages, natural killer (NK) cells, and neutrophils [28]. This arm of the immune system conducts immune surveillance at the early stages of neoplasia and, in principle, eliminates abnormal cells before tumor formation [29]. Conversely, adaptive immunity is antigen-specific and acquires immunological memory after initial exposure to the threat, mounting enhanced, antibody-based responses to any future encounters [30]. Adaptive immunity is carried out by T cells and B cells. Effective cross-talk between these two arms of the immune system involves antigen-presenting cells (APCs; macrophages, DCs, B cells), which uptake non-self antigens from the TME and educate effector T cells to carry out antigen-specific tumor elimination [31]. Together, these innate and adaptive immune cells form the basis of host defense and cancer immune surveillance. 

The significance of the local immune landscape in governing tumor progression and response to therapies has been well-established. It is believed that tumor cells engage in a dynamic, reciprocal interplay with the TME, a complex entity composed of tumor cells, non-neoplastic immune cells, blood vessels, and the extracellular matrix (ECM) [32,33]. The overall composition and phenotype of immune cells within the TME can tip the scale in determining tumor progression versus elimination. Immunologically “cold” tumors are generally characterized by sparse tumor-infiltrating lymphocytes (TILs) and abundant immunosuppressive cells, including tumor-associated macrophages (TAMs) and regulatory T cells (Tregs), making them refractory to ICIs [34,35,36]. Conversely, immunologically “hot” tumors display high TILs and low levels of immunosuppressive cells, rendering them more sensitive to ICIs [37,38]. Therefore, the dominant phenotype of the TME is a critical variable in predicting the success of ICIs in treating a specific cancer. 

Additionally, it has become increasingly apparent that effective tumor eradication is also predicated on the systemic immune landscape beyond the local TME. Peripheral T-cell populations have been shown to sustain the anti-tumor response even after immune cells in the TME have returned to their baseline activity [39]. Therefore, disrupting these systemic cell populations can abrogate the response to ICIs, resulting in systemic lymphodepletion and crippling long-term immune memory [40]. By contrast, promoting effector and memory T-cell development in the peripheral immune system has been found to enhance ICI efficacy in tumor-bearing mice [41], further demonstrating that locally and systemically engaged immunity are equally essential for the overall anti-tumor response. 

Immunotherapy has shown promising results in many devastating cancers but is critically dependent on the immune system’s natural role in cancer surveillance and elimination. Given that ICIs function by disinhibiting the natural immune response against tumor cells, local and systemic immunosuppression are critical barriers to improving patient outcomes through immunotherapy. For these reasons, glioblastoma is an exemplary case of a tumor that employs immune escape in both the local TME and the peripheral immune system and remains a compelling arena for further investigation. 

### 3.2. Glioblastoma Achieves Immune Escape in the Local Tumor Microenvironment

**Figure 1:**
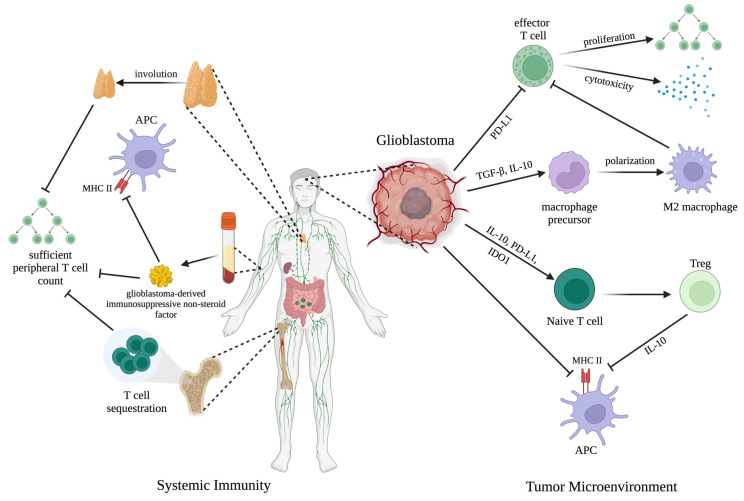
Key glioblastoma immune escape mechanisms mentioned in this review. The efficacy of immune checkpoint inhibitors (ICIs) and chimeric antigen receptor (CAR) T-cell therapy is critically dependent on an intact host immune system in both the tumor microenvironment (TME) and systemic landscape. Glioblastoma fosters an immunosuppressive environment by targeting multiple aspects of host immunity that typically should prevent cancer progression. In the tumor microenvironment, glioblastoma interferes with antigen presentation, promotes M2 macrophage polarization and Treg formation, and directly impedes T-cell cytotoxicity and proliferation. In systemic immunity, glioblastoma is associated with thymic involution and T-cell sequestration in the bone marrow. An immunosuppressive non-steroid factor further disrupts peripheral T-cell function and also impairs antigen presentation. Mechanisms of action are further described in the respective sections of this review. This figure was created with BioRender.com, access date 15 November 2023.

The interaction between glioblastoma cells and their local microenvironment is known to steer tumor development and therapy resistance. Specifically, glioblastoma co-opts multiple local aspects of innate immunity, including disrupting antigen presentation, altering macrophage polarization, and cultivating an inherently immunosuppressive TME. In a functional immune system, pre-cancerous cells are detected via neoantigen presentation on their major histocompatibility complex (MHC) class I molecules, resulting in targeted removal by circulating natural killer (NK) or CD8+ T cells [42]. Increasing evidence has suggested that glioblastoma cells themselves may downregulate MHC class I expression, avoiding this neoantigen presentation and thereby escaping detection by the host’s innate immune system [43]. Loss of MHC class II, which is critical for antigen presentation between APCs and T cells, has also been documented in glioblastoma, speaking to the extensive immunosuppressive effects of this tumor [44] (Figure 1). 

During tumor progression, glioblastoma cells also release unique immunomodulatory factors to modify the neighboring immune microenvironment and selectively enrich certain immune cell phenotypes. In doing so, glioblastoma cells can recruit surrounding innate immune cells to establish a microenvironment conducive to malignancy. TAMs are a major non-neoplastic constituent of glioblastoma tissue and generally display a range of differentiation [45]. Pro-inflammatory (M1) macrophages are classically activated and produce proinflammatory cytokines to initiate the host immune response [46]. On the other hand, M2 macrophages are associated with immunosuppression and tissue repair and have been shown to engage in reciprocal interactions with tumor cells in the TME, facilitating tumor progression [47]. Increasing evidence has suggested that the TAM phenotype is highly plastic and responsive to environmental cues [48]. Glioblastoma exploits this malleability by enriching the local TME with immunomodulatory factors such as transforming growth factor β (TGF-β) and interleukin-10 (IL-10), which polarize TAMs to their immunosuppressive phenotype and reduce their antigen-presenting capabilities [43,49] (Figure 1). 

Certain mechanisms are known to modulate glioblastoma’s ability to functionally polarize innate immune cells to their immunosuppressive phenotype and hamper downstream anti-tumor immunity. For example, increasing evidence has implicated tumor-derived extracellular vesicles (EVs) in glioblastoma immune escape. EVs are small lipid bilayer-encapsulated particles that are shed in large quantities by tumor cells, and have been found to induce immunosuppressive monocytes, including myeloid-derived suppressor cells (MDSCs) [50,51]. Recently, Liu and colleagues have elucidated a complex relationship among programmed cell death (PCD) processes, the immunosuppressive TME, and immunotherapy resistance in glioblastoma [52]. They demonstrated that during a specific type of PCD called ferroptosis, glioblastoma cells release abundant immunosuppressive mediators and chemoattractant factors to promote recruitment and polarization of TAMs into the M2-like immunosuppressive state. Upregulated ferroptosis was associated with attenuated antitumor cytotoxic killing and reduced survival in glioblastoma-bearing mice. 

The above-described changes to innate immunity in the TME directly limit T-cell infiltration into and activation in the tumor bed [53]. Through the release of interleukin-6 (IL-6) and the surface expression of programmed death ligand-1 (PD-L1) and indoleamine 2,3-dioxygenase (IDO) 1, glioblastoma cells induce the recruitment and expansion of Tregs [43] (Figure 1). Tregs then release immunosuppressive cytokines such as IL-10 to further blunt the function of APCs and dampen effector T-cell proliferation and cytotoxicity [43], and they are often associated with a poor cancer prognosis [54] (Figure 1). Additionally, PD-L1 on glioblastoma cells directly promotes effector T-cell exhaustion and anergy [55], resulting in a sparse distribution of CD8+ cytotoxic T cells being detected in the glioblastoma parenchyma, with those present often being refractory to direct T-cell-receptor stimulation [56] (Figure 1). This defective T-cell-mediated cytotoxicity has also been attributed to expression of the inhibitory CD161 receptor, which is hypothesized to interact with programmed cell death (PD-1) expression on T cells [57]. Immunosuppressive TAMs assist tumor cells in enforcing T-cell exhaustion, demonstrating the intimate relationship between the integrity of the innate immune response and the resultant adaptive immune response (Figure 1). While glioblastoma EVs have not been found to directly inhibit T-cell activation, they induce MDSCs which are known to inhibit T-cell proliferation in vitro [50]. Finally, in terms of tumor access, IL-6 has been described as the key glioblastoma-derived factor that promotes anti-inflammatory and wound-healing type responses, inducing strategic tissue remodeling to prevent immunologic access to the tumor by T cells [43]. 

Eventually, the equilibrium between immunologic control and tumor cell proliferation disintegrates, allowing tumor growth to proceed relatively unchecked. This stage of “immune escape” reveals the expansive failure of the innate and adaptive immune responses in glioblastoma. Since current ICI strategies largely rely on sufficient effector T-cell infiltration and activation to exert anti-tumor activity [58], overcoming local immune dysfunction at the tumor bed is a critical prerequisite to successful immune checkpoint inhibition against glioblastoma.

### 3.3. Glioblastoma Induces Widespread Systemic Immunosuppression

Analysis of clinical samples has revealed that in addition to local immunosuppression, glioblastoma patients also exhibit profound systemic immunosuppression even in the absence of metastatic disease. Glioblastoma patients can present with reduced MHC II expression on circulating monocytes, reflecting expansive impairment in the antigen presentation system [59] (Figure 1). 

Additionally, glioblastoma is known to deplete systemic T cells, both in terms of number and anti-tumor activity. Patients often present with low CD4+ T-cell counts comparable to individuals with acquired immune deficiency syndrome and smaller secondary lymphoid organs [60,61]. This paucity of systemic T cells may be explained by glioblastoma-induced loss of sphingosine-1-phosphate receptor 1, which disrupts lymphocyte trafficking and results in naive T cells being sequestered in the bone marrow [60] (Figure 1). Sequestration of T cells may result in antigenic ignorance, thus limiting their anti-tumor potential [62]. Glioblastoma-induced thymus involution has been shown to further dysregulate T-cell development, resulting in a lack of adequate peripheral T-cell replenishment for the host immune response [63] (Figure 1). Mechanistically, MDSCs have been identified in both the tumor bed and peripheral circulation of glioblastoma patients and may negatively influence T-cell maturation and activation in secondary lymphoid tissues [64]. 

Furthermore, the T cells that are present in the systemic environment often lack responsiveness to antigenic stimulation. Peripheral T-cell exhaustion has been linked to a circulating non-steroid factor in serum that directly disrupts the proliferation and function of T cells [63] (Figure 1). Exposure to the serum of a glioblastoma-bearing mouse was sufficient to induce thymic involution and reductions in T-cell counts and MHC II expression in tumor-free mice, revealing the expansive consequences of glioblastoma-induced immunosuppression [63] (Figure 1). 

Therefore, since local immunity relies intimately on peripheral immune cells to drive and enforce the antitumor response [65], developing therapeutic strategies to intervene at the systemic level is equally critical for improving the efficacy of ICIs against glioblastoma.

### 3.4. Potential Combined Approaches to Overcome the Limitations of ICIs in Glioblastoma 

In summary, glioblastoma’s unique mechanisms of immunosuppression present a distinct challenge to current ICI strategies, since these therapies rely on an intact underlying immune system. Specifically, since glioblastoma is known to promote suppressive macrophages and impaired T cells, blockade of a single immune checkpoint may not be sufficient to restore effective anti-tumor activity across the local and systemic spectra. Rectifying this local and systemic immunosuppression may provide a key opportunity for intervention in glioblastoma.

A combinatory approach using current ICIs and concomitant therapies that either reverse macrophage-mediated T-cell immunosuppression or directly improve T-cell activation and proliferation has shown promising results in preclinical studies. Given that ferroptosis has been implicated in glioblastoma immunosuppression and tumor progression, Liu and colleagues found that combined treatment with a ferroptosis inhibitor and PD-1/PD-L1 blockade reversed the immunosuppressive phenotype in tumor-bearing mice, resulting in prolonged survival time and reduced tumor size compared to treatment with PD-1/PD-L1 blockade alone [52] (Table 1). Mechanistically, this dual treatment re-programmed macrophages into the M1 pro-inflammatory state and rescued cytotoxic T-cell activity relative to PD-1/PD-L1 blockade alone. Importantly, ferroptosis inhibition in the absence of PD-1/PD-L1 blockade was also not sufficient to improve the number and function of T cells, suggesting that inhibitory checkpoints can still “brake” T-cell anti-tumor immunity even after M2 polarization has been addressed. Therefore, combining ferroptosis inhibition with ICIs may yield better results than either administered alone. In a similar approach, Yang and colleagues targeted intratumoral immune escape in glioblastoma with the small molecule toosendanin, which was previously shown to reprogram tumor-educated macrophages and rescue T-cell infiltration and activation [53]. In tumor-bearing mice, combination therapy with both toosendanin and ICI (PD-1 and CTLA-4 blockade) outperformed toosendanin or ICI administered alone, slowing tumor growth and improving median survival [53] (Table 1). Their results indicate that an adjunctive treatment that directly addresses immune escape mechanisms in glioblastoma can improve the efficacy of ICI. 

In addition to focusing on the immunosuppressive role of innate immune cells in glioblastoma, contemporary studies that pair ICIs with a therapy that directly addresses adaptive immunity have also yielded positive results in vivo. Zhou and colleagues developed a nanostructure called Nano-reshaper that directly improved T-cell activation, proliferation, and infiltration in glioblastoma, addressing both local and systemic immune dysfunction [66]. This nanostructure co-delivered two therapeutic agents, the small-molecule compound cannabidiol (CBD) and the cytokine LIGHT, which were separately shown to alleviate systemic lymphopenia in vivo and normalize intra-tumoral vasculature, respectively [66]. First, they demonstrated that Nano-reshaper remodeled the local glioblastoma microenvironment, rectifying abnormal blood vessel structure and decreasing levels of glioblastoma-derived immunosuppressive cytokines such as TGF-β and IL-10, resulting in lower levels of Tregs and M2 phenotype TAMs [66]. Systemically, pairing this nanostructure with ICI elevated thymus and spleen indices and demonstrated improved peripheral lymphocyte proliferation [66] (Table 1). Additionally, combining this nanostructure with ICI enhanced long-term survival in tumor-bearing mice and established immunological memory to prevent recurrence. 

In summary, pairing current ICI strategies with therapies that reprogram crucial aspects of local or systemic immune function may provide a clinically translatable opportunity to overcome glioblastoma immune escape and advance the therapeutic efficacy of ICIs for these tumors. 

## 4. Glioblastoma Demonstrates Multiple Mechanisms of Resistance to CAR T-Cell Therapy

### 4.1. Glioblastoma-Induced Immunosuppression and Tumor Heterogeneity Limit the Efficacy of CAR-T Therapy

The above-described immune escape mechanisms that limit the efficacy of ICIs for glioblastoma also complicate the use of CAR T-cell therapy. For example, glioblastoma-derived immunomodulatory factors such as IL-6 and TGF-β have been implicated in the direct impairment of T-cell function and thus may limit the successful implementation of CAR-T therapy [68,69]. Additionally, this tumor is known to exhibit a high degree of heterogeneity in antigen expression and driver mutations, both within the same tumor and across tumors in different patients [70,71]. Transcriptomic diversity constitutes an additional layer of glioblastoma’s characteristic heterogeneity. It has been demonstrated that glioblastoma cells can exist in different cellular states, and genetic alterations in different genes including *CDK4*, *PDGFRA*, *EGFR*, and *NF1* will each favor a distinct state [72]. Furthermore, these states are dynamic and can be altered by the TME [73]. As a result of this significant intratumoral heterogeneity, designing CAR T cell lines that are effective against the entirety of the tumor is a daunting task, and treatment may achieve little more than clonal selection for untargeted mutations [74]. 

### 4.2. Physical Parameters and Features of Glioblastoma Present Additional Challenges to Effective CAR-T Therapy

In addition to immunomodulatory considerations, glioblastoma’s resistance to CAR T-cell therapy may be partially attributed to unique physical barriers that limit the successful distribution and subsequent delivery of adoptive cellular transfer therapy. Given that CAR-T therapy is administered via an infusion into the patients’ bloodstream where the CAR T cells must travel to reach the cancer cells, the distribution of this therapy within the human body may be affected by barriers to access in the central nervous system (CNS) and the tumor’s aberrant vasculature. In vitro studies have demonstrated that human CD4+ T cells may enter the CNS through the blood–brain barrier (BBB) or the blood–cerebrospinal fluid barrier (BCSFB), with different T-cell subsets preferentially crossing one or the other under inflammatory vs non-inflammatory conditions [75]. Clearly, a CAR T-cell therapy targeting glioblastoma would need to be able to consistently enter the CNS through the bloodstream.

Glioblastoma is characterized by extensive, abnormal vasculature that not only sustains the tumor but also allows for its growth and resistance [76]. Glioblastoma stem cells (GSCs) reside in hypoxic perivascular niches that have recently been identified to be primarily periarteriolar instead of pericapillary [77,78]. This distinction between periarteriolar and pericapillary is immunologically relevant, as leukocytes preferentially extravasate through the walls of smaller, thinner vessels, such as capillaries and post-capillary venules [79]. Therefore, glioblastoma’s jungle-like vasculature with predominantly periarteriolar niches may compromise the effective and efficient distribution of CAR T-cell therapy. GSCs are also known to produce and secrete neurotransmitters such as histamine to establish a pro-angiogenic tumor microenvironment, and this aberrant angiogenesis may be reinforced by phosphoglycerate dehydrogenase (PHGDH)-mediated endothelial cell metabolism [67,80]. The PHGDH-mediated pathway promotes endothelial cell overgrowth, with a resulting immune-hostile microenvironment that limits T-cell infiltration into the tumor bed and subsequent activation. PHGDH ablation was shown to prune aberrant tumor vessels in vivo, allowing for improved infiltration and activation of T cells [67].

Other pathways, such as IL-6, that may enhance the growth and invasion of glioblastoma and also limit T-cell infiltration, are worthy of further research [81]. In addition to the aforementioned pathways, optimization of CAR T-cell therapy for glioblastoma also needs to account for the presence of other immune cells that may suppress exogenous T-cell activity [82]. Prior studies have reported that other immune cells, such as neutrophils, macrophages, and other myeloid-derived cells, infiltrate glioblastoma tissue more than T cells do, and these immune cells may interfere with the efficacy of CAR T-cell therapy [83,84,85]. In particular, neutrophils are known to infiltrate glioblastoma tissue in substantial numbers and may enforce aberrant angiogenesis [86].

Sufficient adhesion of CAR T cells to tumor cells is a critical physical parameter of this immunotherapeutic agent. A CAR T-cell therapy used to treat glioblastoma requires a tumor-specific surface target. Finding the optimal antigens for glioblastoma has proven challenging due to the heterogeneous spatial and temporal expression of glioblastoma antigens, which reflects this tumor’s underlying heterogeneity at both the molecular and histological levels [46,87]. In addition, glioblastoma can actively reduce CAR T-cell binding, which decreases productive cytotoxicity through the loss of the interferon-γ receptor (IFNγR) signaling pathway [88]. The loss of this pathway permits glioblastoma to evade immune cell adhesion and become insensitive to CAR T-cell therapy, though this loss in liquid tumors does not convey the same CAR T-cell resistance.

To date, the FDA-approved CAR T-cell therapies have utilized CD19, a primarily B-cell-specific surface antigen, and/or the B-cell maturation antigen found in multiple myeloma, as targets [89]. However, CD19-directed therapy has been shown to cause considerable neurotoxicity in a subset of patients due to certain brain cells also expressing CD19 and thus becoming off-tumor targets [90]. Documented toxicities associated with CAR T-cell therapy also include cytokine release syndrome (CRS) and immune-effector-cell-associated neurotoxicity syndrome (ICANS) [91]. Therefore, further research into this area should maximize the adhesion of CAR T cells to glioblastoma while minimizing undesirable side effects.

### 4.3. Potential Combined Approaches to Overcome the Limitations of CAR T-Cell Therapy in Glioblastoma

As described above, glioblastoma poses significant challenges to the successful delivery of CAR T cells due to significant vascular changes that support the continued proliferation of tumor cells [76]. It was previously thought that the infiltrative nature of glioblastoma would lead to relatively uniform disruption of the BBB and therefore facilitate delivery of CAR T cells to the tumor. However, recent data by Sarkaria and colleagues have demonstrated that the BBB remains intact within some regions of the tumor, which may further complicate CAR T-cell delivery [92]. Therefore, additional methods of delivery must be evaluated to maximize the potential benefits of CAR-T therapies in this disease. One proposed method to improve CAR-T delivery is to infuse CAR T cells intracranially. A study by Brown and colleagues was the first to assess the feasibility of this approach, using IL-13 receptor α2 (IL13Rα2)-directed CD8+ T cells to treat locally recurrent glioblastoma [93]. A number of ongoing clinical trials are further evaluating this approach in primary CNS malignancies expressing human epidermal growth factor type 2 (HER2), including NCT01109095, NCT02442297, and NCT03500991 [94]. Addressing the underlying mechanisms that promote glioblastoma’s immune-hostile vascular microenvironment is another crucial prerequisite to progress. After establishing that PHGDH drives glioblastoma’s jungle-like vasculature and suppressive T-cell immunity, Zhang and colleagues demonstrated that PHGDH inhibition sensitized glioblastoma to CAR T-cell therapy and substantially increased survival in mouse models [67] (Table 1). These results suggest that combining CAR T-cell therapy with PHGDH inhibition may provide a unique opportunity to circumvent glioblastoma immune escape and improve immunotherapy for these tumors.

Establishing the appropriate targets to optimize CAR T-cell binding to glioblastoma remains another priority. As noted above, HER2-directed CAR T cells are being actively evaluated in CNS tumors. A number of additional targets, including IL13Rα2, human epidermal growth factor receptor variant III (EGFR vIII), CD70, CD133, B7H3, and ganglioside (GD2), are all subjects of ongoing investigations [94]. Of these potential targets, IL13Rα2 has been one of the most extensively studied and may be a promising candidate due to the fact that it is expressed at much higher levels in glioblastoma compared to normal brain cells [95]. Additionally, future research efforts should remain mindful of the structural biology underlying CAR T-cell killing to enable optimal treatments. Given that the loss of IFNγR1 signaling has been implicated as major mechanism of glioblastoma-intrinsic resistance to CAR T cells, successful immunotherapy requires maintenance of the physical mechanics of cytotoxicity and an adequate synapse between tumor and CAR T cells [88]. 

In summary, identifying the optimal route for CAR T-cell delivery, addressing the underlying biological mechanisms for aberrant vascularization, and verifying the optimal antigenic targets remain three of the most important areas of ongoing research in treating glioblastoma. Addressing these questions may allow clinicians to fully unlock the potential benefits of CAR T-cell therapy in glioblastoma that have been documented for liquid tumors.

## 5. Conclusions

This review offers an overview of the current understanding of immune escape mechanisms in glioblastoma as they relate to and directly impede current immunotherapy efforts. Agents such as ICIs and CAR-T therapy have demonstrated tremendous success in many tumors that were previously refractory to standard treatments. Despite these encouraging results, similar efforts for glioblastoma have thus yielded disappointing outcomes. 

Several challenges across both the local and systemic immune landscapes currently limit the efficacy of ICIs and CAR T-cell therapy in treating glioblastoma. It has become clear that glioblastoma not only interferes with multiple aspects of host immunity but also utilizes abnormal vasculature and antigen heterogeneity to circumvent current therapies. 

Combination therapy with current immunotherapeutic agents and agents that directly address mechanisms of immune escape in glioblastoma have demonstrated promising preclinical results in mouse models and may offer a new approach for overcoming glioblastoma’s resistance to immunotherapy. Future investigations should verify the efficacy of combination treatment with these agents in immune-humanized patient-derived xenograft models to inform safety and applicability for clinical treatment in humans. Additional molecular entities with the potential to reverse critical elements of glioblastoma immunosuppression should be explored for their therapeutic potential. Further elucidation of the immune pathways that are hijacked or modified in glioblastoma will contribute to defining future opportunities for immunotherapeutic intervention for patients with this devastating tumor.

## Figures and Tables

**Table 1:** Preclinical results of immunotherapy combined with agents that reprogram immune function mentioned in this review. ↑ = increased, ↓ = decreased.

Adjunctive Treatment	Mechanism of Adjunctive Treatment	Results
Ferroptosis inhibitor (Ferrostatin-1)	↑ M2 to M1 polarization ↑ T-cell activity	Combined treatment with a ferroptosis inhibitor and PD-1/PD-L1 blockade reversed the immunosuppressive phenotype in glioblastoma-bearing mice, resulting in prolonged survival time and reduced tumor size compared to treatment with PD-1/PD-L1 blockade alone or ferroptosis inhibition alone [52].
Small-molecule toosendanin	↑ M2 to M1 polarization ↑ T-cell infiltration and activation ↓ T-cell exhaustion	a. Combined treatment with toosendanin and ICIs (anti-PD-1 and anti-CTLA-4 antibodies) delayed tumor growth and enhanced mouse survival compared to treatment with toosendanin or ICIs alone [53]. b. Combined treatment with toosendanin and CAR T-cell therapy significantly enhanced mouse survival compared to treatment with toosendanin or CAR T-cell therapy alone [53].
Nanostructure Nano-reshaper	↑ number of systemic T cells ↑ local T-cell recruitment ↑ APC activity ↑ normalization of blood vessels ↑ M2 to M1 polarization	Combined treatment with Nano-reshaper and PD-1 blockade improved long-term survival in glioblastoma-bearing mice and generated immunological memory to prevent recurrence [66].
PHGDH inhibition	↓ aberrant vessel sprouting ↓ intratumoral hypoxia ↑ T-cell infiltration and activity	Combined treatment with PHGDH inhibition and CAR T-cell therapy improved overall survival and delayed tumor growth compared to treatment with PHGDH inhibition or CAR T-cell therapy alone [67].

PD-1, programmed cell death protein 1; PD-L1, programmed cell death ligand 1; ICI, immune checkpoint inhibitor; CTLA-4, cytotoxic T-lymphocyte-associated protein 4; CAR, chimeric antigen receptor; APC, antigen presentation cell; PHGDH, phosphoglycerate dehydrogenase.

## Data Availability

Not applicable.
